# Atypical Takotsubo Cardiomyopathy Presenting as ST-Elevation Myocardial Infarction

**DOI:** 10.7759/cureus.90250

**Published:** 2025-08-16

**Authors:** Sana Tariq, Hazem Elshenawy

**Affiliations:** 1 Cardiology, Manchester University National Health Service (NHS) Foundation Trust, Manchester, GBR; 2 Acute Medicine and Cardiac Intervention, Manchester University National Health Service (NHS) Foundation Trust, Manchester, GBR

**Keywords:** apical sparing, focal takotsubo cardiomyopathy, normal coronary arteries, stemi, transient left ventricular dysfunction

## Abstract

A 64-year-old woman with a past medical history of diverticulosis and duodenal ulcer presented with classical features of ST-elevation myocardial infarction (STEMI), including cardiac sounding chest pain, ST-elevations on electrocardiography (ECG), and elevated cardiac biomarkers. She was an ex-smoker and had a family history of heart disease, but did not report other significant cardiovascular risk factors such as hypertension, diabetes, or hyperlipidemia. Notably, she also described experiencing a recent period of emotional stress due to her friend being diagnosed with cancer. The case was initially considered a routine presentation of STEMI. However, on the day of admission (day 0), a coronary angiogram was performed, which revealed unobstructed coronary arteries. The left ventricular angiogram demonstrated mid-ventricular ballooning with a normal apex. Subsequent imaging included an echocardiogram on day 1, which showed a normal left ventricular size but impaired systolic function with ejection fraction (EF) of approximately 45% and regional wall motion abnormalities. A cardiac magnetic resonance imaging (MRI) scan performed on day 5 showed normal cardiac size and function with hypokinesia in the mid- to apical anteroseptal and anterior wall regions and a normal apex, along with mild edema, patchy diffuse late gadolinium enhancement, and elevated T1 values. These imaging features were consistent with atypical Takotsubo cardiomyopathy (TTC) or regional myocarditis. The improvement in EF from 45% to 63% over a few days, along with the absence of viral prodrome and pericardial effusion, indicates reversible ventricular dysfunction, further supporting the diagnosis of TTC.

She showed significant improvement during her hospital stay, with left ventricular function returning to normal. She was started on a beta-blocker, and we advised her general practitioner (GP) to prescribe ramipril once her blood pressure improves. She was followed up at her local hospital and has returned to her usual daily activities.

This case underscores the importance of considering stress-induced cardiomyopathy in differential diagnosis when patients present as acute coronary syndrome but have normal coronary arteries. While typical TTC is characterized by apical ballooning, atypical variants such as basal hypokinesia, mid-ventricular hypokinesia, and isolated right ventricular involvement can occur, often presenting with variable echocardiographic findings. In this patient, regional wall motion abnormalities were seen without typical apical involvement. Although B-type natriuretic peptide (BNP) was not measured in this case, a relatively high BNP-to-troponin ratio is seen in TTC due to marked ventricular wall stress. If this had been measured in our patient, it might have facilitated a quicker diagnosis and steered the management strategy away from ischemic etiologies. This will be considered in future similar cases to support earlier diagnosis and management.

## Introduction

Takotsubo cardiomyopathy (TTC) is a transient and reversible ventricular dysfunction syndrome usually precipitated by emotional or physical stress and predominantly affects women. It closely mimics acute coronary syndrome (ACS), presenting with chest pain, electrocardiographic (ECG) changes, and elevated cardiac biomarkers, but is distinguished by unobstructed coronary arteries on angiography. Approximately 1%-2% of patients initially diagnosed with ACS are ultimately found to have TTC [1]. One of the underlying mechanisms is thought to be adrenergic hyperactivity in response to stress [2]. Alternatively, a clinical trial demonstrated endothelial dysfunction as a possible mechanism involved in the pathophysiology of TTC [3]. Both of these mechanisms can trigger coronary artery spasm, leading to myocardial ischemia. Physical stress is more frequently implicated in male patients, while emotional triggers or no identifiable stressors are more common in female patients [4].

The classical form of TTC is characterized by apical ballooning with basal segment hyperkinesis. Atypical variants of TTC differ in the distribution of wall motion abnormalities seen on echocardiography or cardiac magnetic resonance imaging (MRI). The mid-ventricular type shows hypokinesia of the mid-ventricular segments with preserved basal and apical function, the basal (reverse) type shows basal hypokinesia with apical hyperkinesia, and the focal type involves isolated dysfunction in a single ventricular segment. A clinical study by Kurowski et al. reported that 60% of TTC patients exhibit typical features, while 40% present with atypical patterns [5].

TTC should be considered a key differential diagnosis in patients presenting with ACS-like symptoms and acute ventricular failure, especially when coronary arteries appear normal. Our case highlights an atypical form of TTC, which is infrequently reported in the literature. While Takotsubo is often described as apical ballooning syndrome, it can present atypically with sparing of the apex. Emphasizing these atypical cases helps refine diagnostic accuracy by challenging traditional presentations and improves clinical decision-making. Additionally, it broadens awareness and education about rare variants of the condition. Other differentials include myocarditis, pericarditis, substance-induced coronary vasospasm, and MINOCA (myocardial infarction with non-obstructive coronary arteries).

## Case presentation

A woman in her early 60s with a history of diverticulosis, duodenal ulcer, and a family history of ischemic heart disease presented with a sudden onset of occipital headache followed by chest pain at rest, radiating to the neck and left arm, which started about 24 hours ago. She was an ex-smoker. She reported significant emotional stress over the preceding two weeks due to a close friend's recent cancer diagnosis.

**Figure 1:**
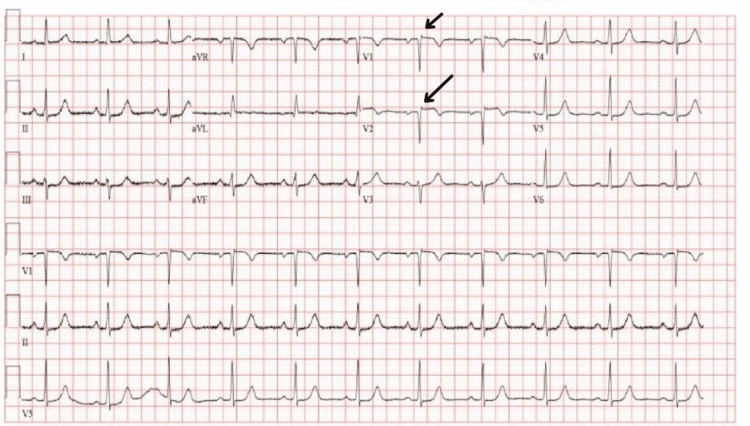
ECG showing anterior ST-elevation, Q waves, and T wave inversion in V1 and V2. Initially suggestive of anterior STEMI, later found to be Takotsubo cardiomyopathy ECG: electrocardiography; STEMI: ST-elevation myocardial infarction

On examination, vital signs were stable, and she was pain-free. ECG demonstrated 1 mm ST-segment elevation, Q waves, and T wave inversions in leads V1-V2 (Figure 1). Her blood tests were otherwise unremarkable, except for significantly elevated cardiac biomarkers. Troponin T was initially measured at 461 ng/L with a subsequent measurement of 113 ng/L taken five hours later (Table 1). A third troponin measurement taken two days after showed a further decrease to 58 ng/L. Based on the clinical picture of typical cardiac sounding chest pain, ischemic ECG changes, and elevated cardiac enzymes, a working diagnosis of late-presentation ST-elevation myocardial infarction (STEMI) was made.

**Table 1:** Blood results eGFR: estimated glomerular filtration rate; HDL: high-density lipoprotein; LDL: low-density lipoprotein

Blood	Test result	Reference range
Hemoglobin	134 g/L	115-165 g/L
White cell count	7.3 × 10⁹/L	4.0-11.0 × 10⁹/L
Creatinine	58 µmol/L	45-84 µmol/L
eGFR	>90 mL/min/1.73 m²	>90 mL/min/1.73 m²
Troponin T (first sample)	461 ng/L	0-14 ng/L
Troponin T (second sample)	113 ng/L	0-14 ng/L
Total cholesterol	5.8 mmol/L	Below 5 mmol/L
HDL	1.3 mmol/L	Above 1.2 mmol/L for women
LDL	3.9 mmol/L	Below 3.0 mmol/L

**Figure 2:**
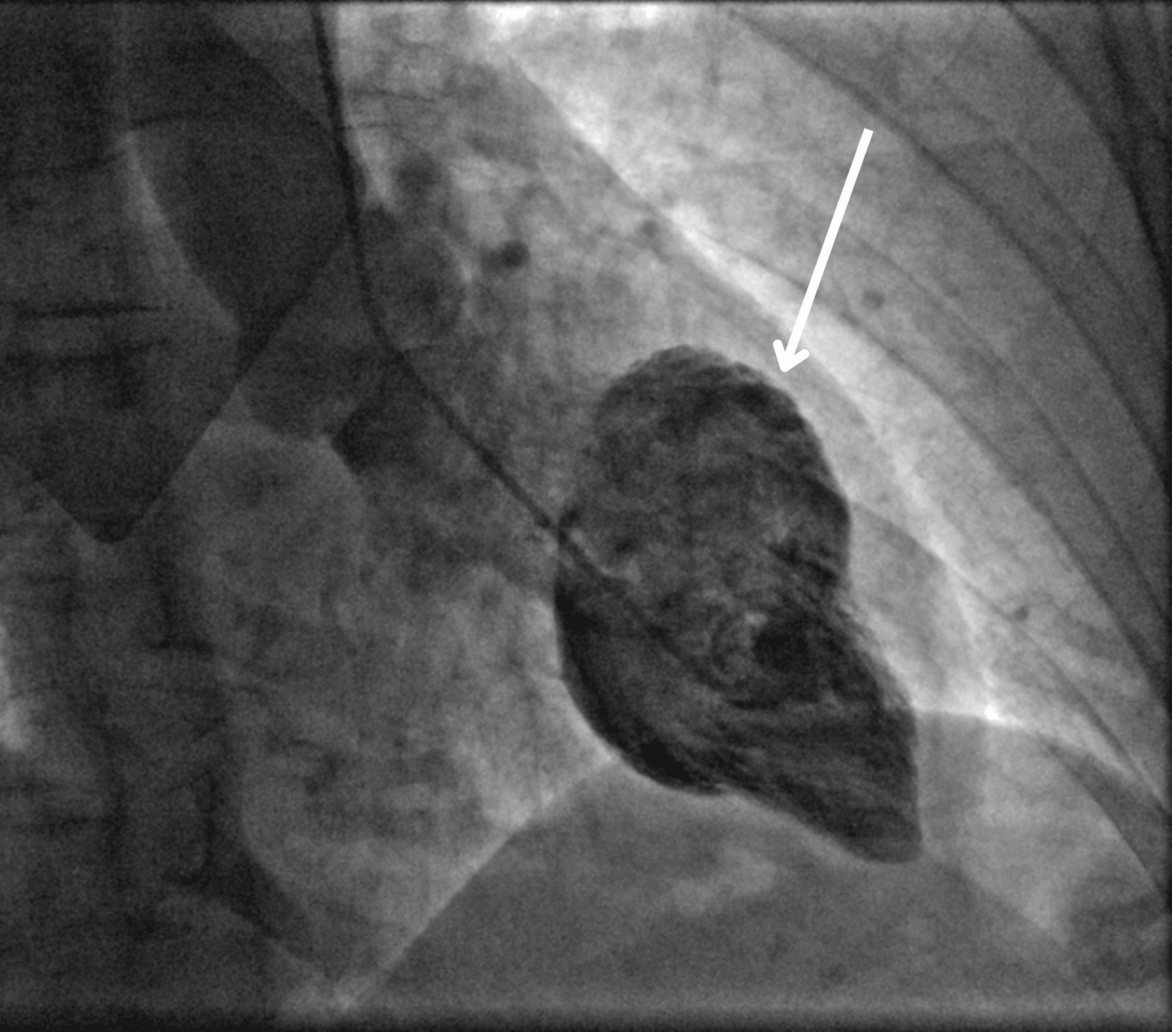
LV angiogram during diastole LV: left ventricular

**Figure 3:**
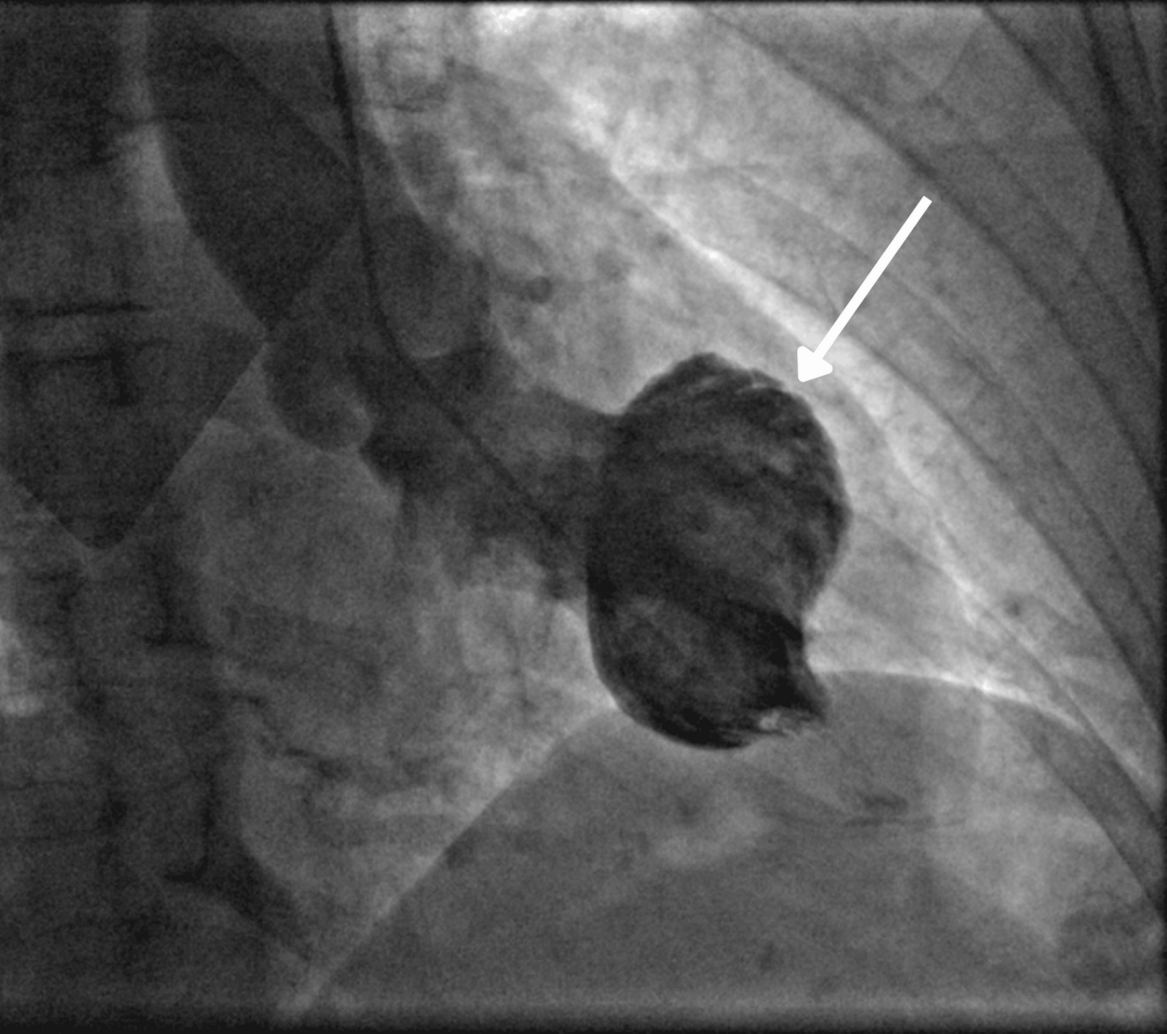
LV angiogram showing apical contraction and mid-ventricular ballooning during systole LV: left ventricular

The patient was taken to the cardiac catheterization laboratory for percutaneous coronary intervention (PCI). Coronary angiography showed normal coronary arteries with a left dominant coronary system. Left ventriculogram revealed impaired left ventricular (LV) systolic function with ballooning of the anterior and inferior walls, sparing the apex (Figures 2, 3).

**Figure 4:**
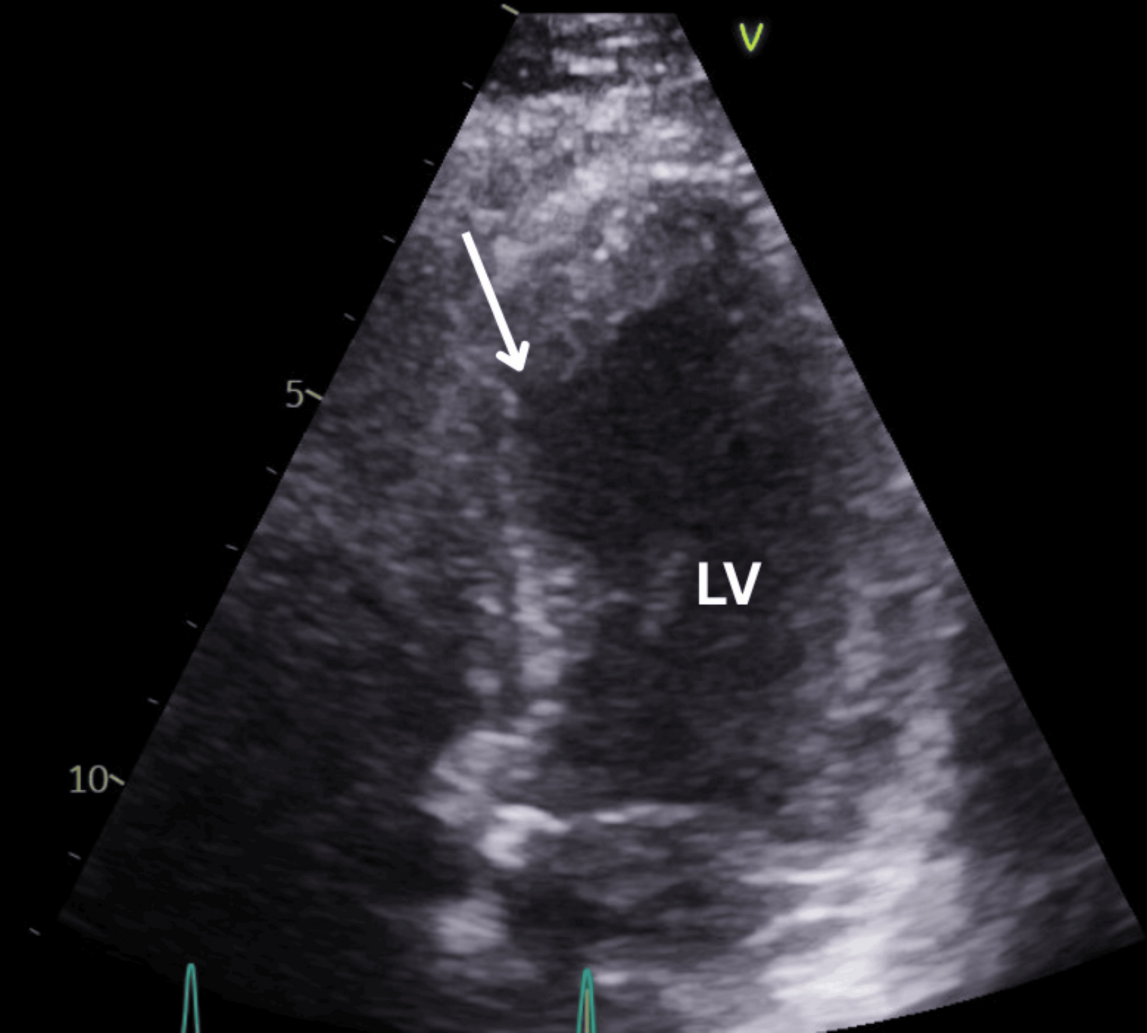
Transthoracic echocardiogram (systolic phase) showing the left ventricle (LV) with mid-septal ballooning, whereas the apex has normal contraction

A transthoracic echocardiogram (TTE) performed post-procedure demonstrated thinning and akinesia of the mid-anteroseptal and infero-septal walls, hypokinesia of the apical septal and mid-anterior walls, and moderately impaired LV systolic function with an estimated ejection fraction of 45% (Figure 4).

**Figure 5:**
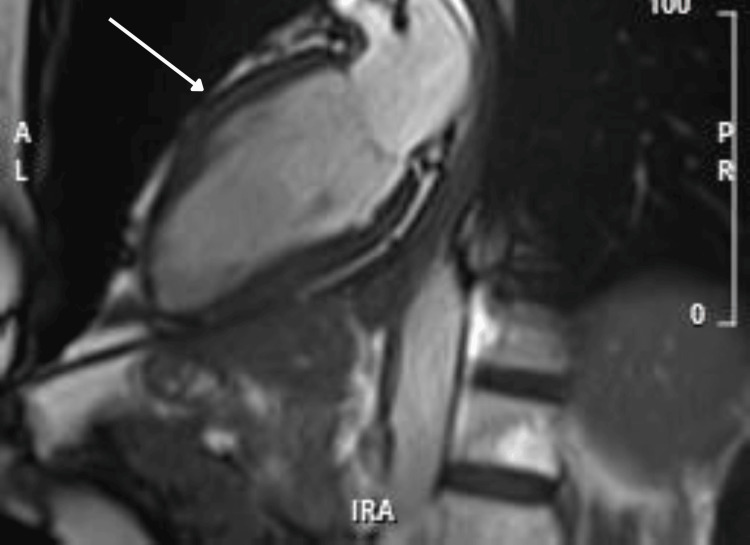
Cardiac MRI showing left ventricular diastole MRI: magnetic resonance imaging

**Figure 6:**
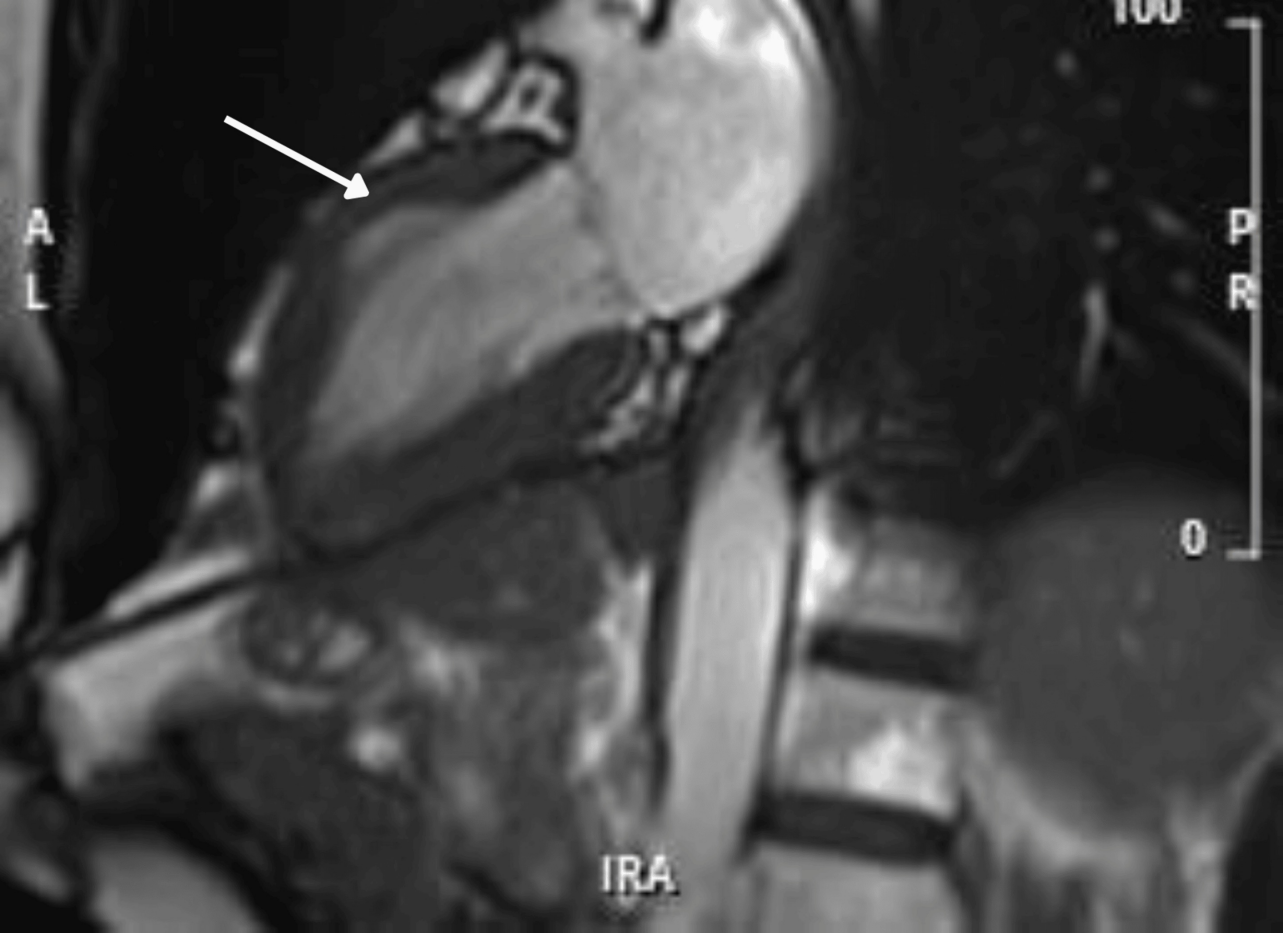
CMR during systole demonstrating mid-ventricular ballooning with apex in contraction phase CMR: cardiovascular magnetic resonance

**Figure 7:**
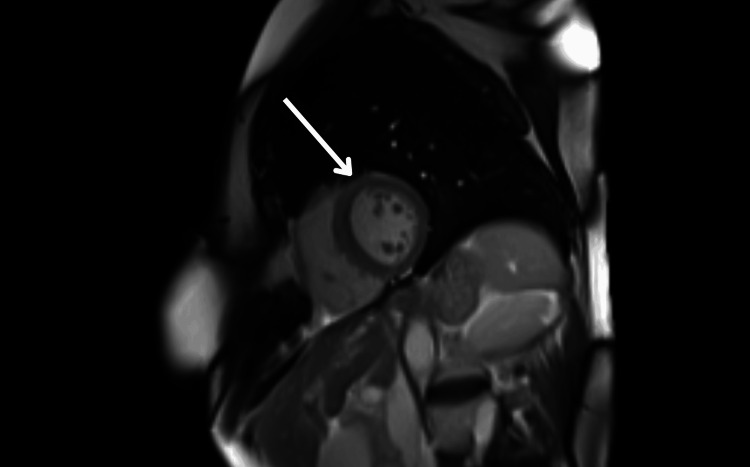
Cardiac MRI demonstrating myocardial inflammation MRI: magnetic resonance imaging

**Figure 8:**
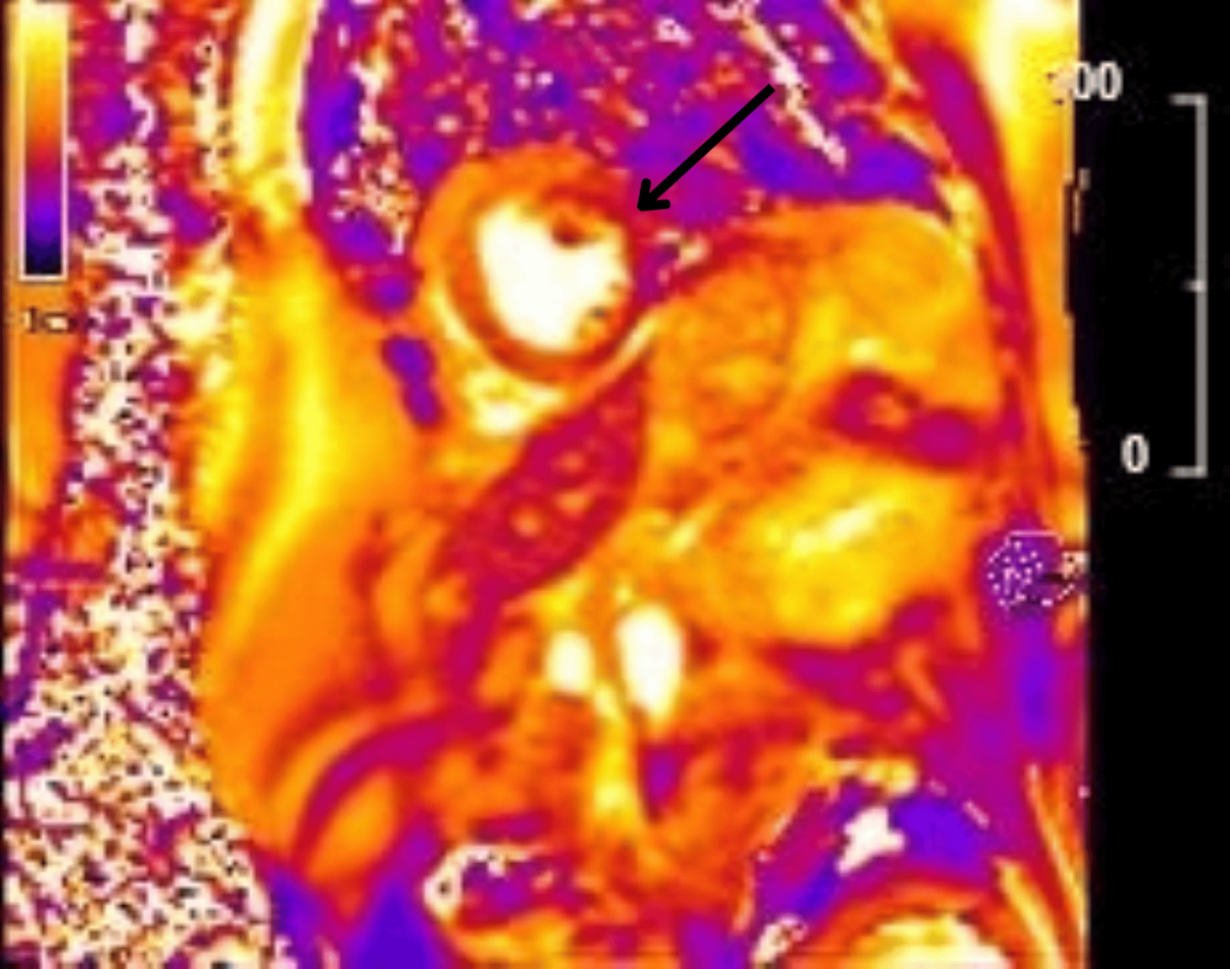
T2 image showing myocardial inflammation

Five days after admission, a cardiac MRI scan was performed to further evaluate myocardial structure and function. MRI findings included normal LV systolic function with an improved ejection fraction of 63%, mild hypokinesia of the mid- to apical anteroseptal and anterior walls, a normal apex with no evidence of ballooning, and mild myocardial edema in affected segments with patchy, diffuse late gadolinium enhancement with elevated native T1 values (Figures 5-8).

This marked improvement in LV function is a hallmark of TTC, where myocardial dysfunction is typically transient and reversible. The rapid recovery observed in this case aligns with the expected course of TTC, further supporting the diagnosis despite the initial presentation mimicking late STEMI. This dynamic change in LV function highlights the characteristic feature of TTC, its reversible nature, distinguishing it from other causes of acute myocardial injury.

These findings confirmed the diagnosis of atypical TTC. Other differential diagnoses considered were myocarditis and MINOCA; however, the absence of recent infections, a rapidly decreasing troponin level, swift recovery of LV function, and the absence of pericardial effusion made these less likely. Additionally, a history of emotional stress preceding symptom onset further supported the diagnosis of TTC.

The patient was initially managed as a late-presentation STEMI case and taken for PCI. Following the discovery of normal coronary arteries, management was adjusted to supportive care for TTC, including beta-blockers. Angiotensin-converting enzyme (ACE) inhibitors were planned to be started as an outpatient if blood pressure was stable. The patient was followed up at her local hospital, and she recovered well.

## Discussion

TTC, also referred to as "broken heart syndrome," is a form of transient ventricular dysfunction most often precipitated by acute emotional or physical stress. Clinically, TTC closely mimics ACSs presenting with chest pain, ECG changes, and elevated cardiac biomarkers. However, coronary angiography typically reveals no significant obstructive coronary artery disease. This condition predominantly affects postmenopausal women and is now recognized as a distinct clinical entity with unique diagnostic criteria and management considerations.

In this case, anterior STEMI was initially suspected due to ECG changes and elevated troponin levels. However, normal coronary angiography and echocardiographic evidence of regional wall motion abnormalities (RWMAs), particularly extending beyond a single coronary territory, supported a diagnosis of TTC. The absence of recent infection, lack of recreational drug use, and presence of emotional stress as a trigger further reinforced this diagnosis. Moreover, the transient nature of LV dysfunction observed in follow-up imaging aligned with the usual course of TTC. In our case, RWMAs were predominantly observed in the mid-ventricular segments, with the apex remaining unaffected. This pattern suggests an atypical form of TTC, as the typical presentation is characterized primarily by apical ballooning, thus making this case unique and highlighting the importance of considering an alternative diagnosis in patients presenting with ACS but having unobstructed coronary arteries.

The pathogenesis of TTC is not fully understood, but it is widely accepted that catecholamine surge plays a critical role. Wittstein et al. demonstrated significantly higher levels of plasma catecholamines in TTC patients compared to those with myocardial infarction (Killip class III), suggesting a neurohumoral mechanism involving myocardial stunning, coronary vasospasm, and microvascular dysfunction [6].

The InterTAK Diagnostic Criteria proposed in the European Heart Journal consensus document offers a standardized framework for diagnosis [7]. Key elements include transient LV dysfunction with RWMAs typically extending beyond a single coronary artery distribution, possible but not obligatory emotional or physical stressor, neurological disorders and pheochromocytoma as potential triggers, new ECG changes such as ST-segment shifts or T wave inversions, moderate elevation of cardiac enzymes and significantly elevated B-type natriuretic peptide (BNP), absence of infectious myocarditis, presence of coronary artery disease not excluding TTC, and a predominance among postmenopausal women. These criteria aim to guide clinicians through the differential diagnosis of TTC. Our patient fit most of the criteria like emotional trigger, ECG changes, elevation of cardiac enzymes, transient LV dysfunction, and absence of prodromal illness.

Several anatomical subtypes of TTC have been described, with apical ballooning being the most prevalent (accounting for 50%-80% of cases) [8]. Other recognized patterns include a basal (inverted) variant demonstrating basal hypokinesia with apical hypercontractility, a mid-ventricular variant having hypokinesia of the mid-LV with sparing of the apex and base, and focal, biventricular, and right ventricular variants. These subtypes highlight the variable patterns of TTC and the importance of careful echocardiographic and cardiac MRI assessment.

Although we did not measure these in our patient, N-terminal prohormone of brain natriuretic peptide (NT-proBNP) and BNP are valuable biomarkers not only for diagnosis but also for prognosis in TTC. A meta-analysis showed that natriuretic peptides were significantly higher in patients with TTC as compared to ACS [9]. A high NT-proBNP-to-troponin T ratio (>7.5) can help differentiate TTC from ACSs with high sensitivity and specificity [10].

Treatment of TTC is largely supportive, focusing on hemodynamic stabilization and addressing potential complications. There are no guidelines based on randomized controlled trials for management. Beta-blockers and ACE inhibitors are commonly used, although their role in preventing recurrence is not definitively established. While the overall prognosis is favorable, rare but serious complications such as cardiogenic shock, LV outflow tract obstruction (LVOTO), and arrhythmias may occur.

Long-term follow-up data reveal a recurrence rate of up to 10% with an annual mortality of approximately 5.6% per patient year and nearly 10% incidence of major adverse cardiac and cerebrovascular events (MACCEs) [11]. These findings emphasize the need for ongoing surveillance and patient education regarding stress management and cardiovascular risk modification.

Several case reports in recent literature describe TTC triggered by various stressors and presenting with ECG patterns like STEMI. To et al. reported a case of a 60-year-old female patient who experienced emotional stress triggered by bereavement. Imaging showed apical ballooning of the heart wall [12]. Yazdi et al. described a 75-year-old previously healthy female patient who developed severely hypokinetic mid-apical segments with preserved contractility of basal segments following a seizure [13]. Lastly, Munshi et al. detailed a case of mid-ventricular TTC presenting as cardiac asthma in a 69-year-old hypertensive female patient, which was managed conservatively [14]. These cases reinforce the varied presentations of TTC and its frequent occurrence in female patients following emotional or physical stressors, with most recovering completely under supportive care.

## Conclusions

This case highlights an atypical presentation of TTC characterized by sparing of the apex. It was successfully distinguished from acute myocardial infarction through imaging and clinical context, particularly noting emotional stress as a triggering factor. This emphasizes the need to consider a broad differential diagnosis in patients presenting with chest pain and ECG changes, especially when initial coronary angiography is normal and there is a history of emotional stress alongside imaging features suggestive of TTC. While TTC typically presents with apical ballooning due to apical hypokinesia, it is important to recognize atypical variants where RWMAs occur but the apex is spared. Given TTC’s reversible nature yet potential for serious complications, awareness and timely diagnosis are crucial in clinical practice. Cardiac MRI plays a key role in confirming the diagnosis, particularly in atypical cases. Although TTC generally has a favorable prognosis with supportive care, follow-up is necessary to monitor for LV function recovery or complications.
